# Commentary: The Embodied Brain: Towards a Radical Embodied Cognitive Neuroscience

**DOI:** 10.3389/fnhum.2015.00623

**Published:** 2015-11-16

**Authors:** Przemysław R. Nowakowski

**Affiliations:** Center for Philosophical ResearchWarsaw, Poland

**Keywords:** embodied cognition, embodied mind, radical embodied cognitive science, embodied cognitive neuroscience, cognitive psychology, ecological dynamical psychology

There is a tension between the embodied approach to cognition (e.g., Varela et al., 1991; Anderson, 2003; Wilson and Foglia, 2011; Myachykov et al., 2014) and the neurocentric way of thinking. It manifests in the research on mirror neurons (Caramazza et al., 2014), action language understanding (Meteyard et al., 2012), or decision making (Kubanek and Snyder, 2015), to mention just a few examples. In spite of radically embodied approaches to neuroscience (Thompson and Varela, 2001; Favela, 2014), the integration of neuroscience and embodiment is still a challenging requirement (Haselager et al., 2008; van Dijk et al., 2008; Gallagher et al., 2013). Kiverstein and Miller's (2015, further: K&M) new paper should be read in that context.

My main concern is that K&M's main argument begs the question. I would summarize it as follows:

1. Emotions are essentially connected with the state of a whole organism and action related.
2. In the brain, emotional, and cognitive processes are tightly interconnected.

Therefore:

3. Cognition is essentially connected with the state of whole organism and action related.

This argument appeals to neuroscience [mainly Pessoa (2014) and Anderson (2014)] to argue for embodiment of cognition^1^, not for embodied neuroscience. Therefore, K&M's approach on embodied cognitive neuroscience (further: ECN) begs the question: i.e., to have embodied neuroscience, we must assume embodiment. But perhaps this assessment is unfair? Notice that there are two issues that K&M deal with: (a) *revision of the division of labor*, and (b) *whole organism and environment determine the function of neural networks*. I start from the second problem.

K&M write:“To determine the precise functional contribution of a network to an animal's behavior we must look at how this network functions in the context of a wider organism-environment system” (p. 2). But what does it mean? Perhaps they mean “the functions a given network performs (…) in (…) a context-dependent manner” (ibid.)? If this is the case, then this is not ECN. Context-dependency is an important feature of cognition, but it's not enough for ECN, and it could be endorsed easily by a more traditional cognitive neuroscience.

The second issue is the revision of division of labor. K&M say that in cognitive neuroscience, we should replace cognitive psychology with dynamic ecological psychology, but this is problematic. The whole idea of the division of labor is now quite contentious (Poldrack, 2010; Anderson, 2015). So we must indicate not only what to replace, and with what, but also how to do it. First, the role of cognitive psychology in cognitive neuroscience may seem problematic (Poldrack, 2010). Second, replacing it with ecological psychology is not an easy task (for further information on its complexity, see: Costall, 2007).

There is yet another problem with this strategy. K&M write: “[c]ognitive neuroscience then seeks to determine how (…) cognitive operations are carried out by brain…” (p. 2). If we apply the same strategy to ECN (as K&M do) and explain phenomena in embodied cognitive science neuroscientifically, we will still discuss embodied cognitive science. We will face the problem of a vanishing difference between embodied cognitive science and ECN, and this is why we should interpret embodiment in neuroscience differently. To defend it, one should first argue for embodied brain functions, and then account for cognitive processes using properly embodied neuroscience.

My argument is not only that brain networks are dynamic and context-dependent, but if neuroscience is embodied, these networks should be extended to non-neuronal body parts^2^. So, not only they are part of a larger system, but they themselves are larger—extended to some bodily (and perhaps environmental) factors. An important starting point might be Wilson and Foglia (2011, W&F) and Wilson and Golonka (2013, W&G). W&G argue that to explain cognition we should focus on a specific task and the resources used during the task. In this context, we can ask: How important is the brain as a resource? What does the brain do when we perform this task? In the case of embodied cognition, one thing is certain—the brain doesn't perform this task alone, and the non-neural body also has a job to do! W&F indicate that during cognition the body can play the role of a distributor, constraint or controller. When the body is a distributor, brain function is replaced by the non-neural body. When the body is a controller, brain activity is modulated, inhibited or amplified by non-neural bodily processes. When the body is a constraint it prevents some cognitive processes (e.g., bodily construed temporal frames for cognitive performances). Obviously, these roles are not mutually exclusive, rather they have constant mutual interaction, amplification, and modulation, to the degree that the role played by the brain in ECN requires a new conceptualization. The brain executes only a part of the cognitive duties, and it is not always the same part (quantitatively and qualitatively).

I think that a good supplement to this line of thinking would be recent works of Pessoa (2014) about *dynamic affiliations* and Anderson (2014) about TALoNS (*Transiently Assembled Local Neural Subsystems*). These studies are not (yet) part of ECN, but they could be. What would be needed for that? i.e., we should extend *dynamical temporal integration* or *dynamic affiliation* to the role played by a non-neural part of body in this process (e.g., self-structuring information, see: Lungarella and Sporns, 2006). Hence, we need “extendedTALoNS”^3^. Such an account will be quite consistent with that proposed by W&G in their views on cognition as a dynamical assemble of resources. And because of a lack of such (or similar) approach in K&M, their main argument begs the question.

## Conflict of interest statement

The author declares that the research was conducted in the absence of any commercial or financial relationships that could be construed as a potential conflict of interest.

## Footnotes

^1^K&M focus on emotion and cognition, but in this commentary I reduce this to the problem of embodied cognition in general.

^2^An interesting new example of such a case is the role of body-environment interaction during catching the ball by an outfielder described by Clark (2015) as a part of primary neuronal process of predictive error minimization.

^3^I think that Clark (2015) describes extendedTALoNS when he describes the outfielder.
